# A Challenging Form of Non-autoimmune Insulin-Dependent Diabetes in a Wolfram Syndrome Patient with a Novel Sequence Variant

**DOI:** 10.4172/2155-6156.1000561

**Published:** 2015-06

**Authors:** Liliana P Paris, Yoshihiko Usui, Josefina Serino, Joaquim Sá, Martin Friedlander

**Affiliations:** 1 Department of Cell and Molecular Biology, The Scripps Research Institute, 10550 North Torrey Pines Road, La Jolla, CA 92037, USA; 2 Ophthalmology Department, Hospital Pedro Hispano, Matosinhos – Greater Oporto area, Portugal; 3 CGC Genetics, Portugal

## Abstract

Wolfram syndrome type 1 is a rare, autosomal recessive, neurodegenerative disorder that is diagnosed when insulin-dependent diabetes of non-auto-immune origin and optic atrophy are concomitantly present. Wolfram syndrome is also designated by DIDMOAD that stands for its most frequent manifestations: diabetes insipidus, diabetes mellitus, optic atrophy and deafness. With disease progression, patients also commonly develop severe neurological and genito-urinary tract abnormalities. When compared to the general type 1 diabetic population, patients with Wolfram Syndrome have been reported to have a form of diabetes that is more easily controlled and with less microvascular complications, such as diabetic retinopathy.

We report a case of Wolfram syndrome in a 16-year-old male patient who presented with progressive optic atrophy and severe diabetes with very challenging glycemic control despite intensive therapy since diagnosis at the age of 6. Despite inadequate metabolic control he did not develop any diabetic microvascular complications during the 10-year follow-up period. To further investigate potential causes for this metabolic idiosyncrasy, we performed genetic analyses that revealed a novel combination of homozygous sequence variants that are likely the cause of the syndrome in this family. The identified genotype included a novel sequence variant in the Wolfram syndrome type 1 gene along with a previously described one, which had initially been associated with isolated low frequency sensorineural hearing loss (LFSNHL). Interestingly, our patient did not show any abnormal findings with audiometry testing.

## Introduction

Wolfram syndrome (WS) is a rare multisystem neurodegenerative disorder of autosomal recessive origin that minimally requires the presence of two diagnostic criteria, insulin-dependent diabetes mellitus (of non autoimmune origin) and progressive optic nerve atrophy [1]. WS is also referred to as DIDMOAD, an acronym for its most common clinical presentation that includes: diabetes insipidus (DI), diabetes mellitus (DM), optic atrophy (OA) and deafness (D) [2].

Even through diabetes mellitus and optic atrophy are the earliest and most common manifestations of WS, neurological and genito-urinary tract complications, which usually develop at later disease stages, are especially concerning, as they constitute the leading causes of morbidity and mortality in the patient population [2,3]. WS is classified into type 1 or type 2, according to the genetic mutation that determines the pathological phenotype.

WS type 1 is caused by mutations in the *WFS1* (Wolfram syndrome type 1) gene and is responsible for approximately 90% of the WS cases worldwide; Incidence is variable depending on geographic location, with reported estimates of 1/700.000 in the UK and 1/100.000 in South America [4]. Even though mutations of exon 8 of the *WFS1* gene (NM_006005; chromosome 4p16.1) cause the majority of WS type 1 cases, this syndrome is characterized by significant genetic heterogeneity, which contributes to a non-linear genotype-phenotype correlation [5,6].

The *WFS1* gene encodes wolframin, a transmembrane protein localized to the endoplasmic reticulum (ER) that is involved in membrane trafficking, secretion, processing and regulation of ER calcium homeostasis, therefore being critical for preventing ER stress signaling [7]. Wolframin is ubiquitously expressed but its highest levels are found in pancreatic beta cells, cardiomyocytes and specific neurons [8]. It has been shown that deletion of the *WFS1* gene in rodents leads to progressive pancreatic beta cell loss due to increased ER stress, along with impaired insulin secretion and higher incidence of diabetes [9–11]. In humans, various genetic studies have also shown a strong association between *WFS1* gene variants and increased risk of type 2 diabetes [12–14]. The existence of *WFS1* variants with different severities, with inactivating or non-inactivating properties, and the way in which these interact to induce and modulate phenotypic expression of progressive pathological features remains unclear.

In this study we identify a novel *WFS1* missense sequence variant in a WS patient and describe its associated progressive clinical picture (over a 10-year follow-up period) in a 16-year old patient who developed an especially challenging form of insulin-dependent diabetes at the age of 6.

## Case Report

A 6-year-old male patient with a history of mild learning disabilities was referred to our hospital for polyuria and polydipsia and diagnosed with insulin dependent diabetes, which rapidly proved to be particularly challenging in terms of metabolic control with fasting blood glucose levels ranging from 203 to 431 g/dl, despite intensive therapy with different therapeutic regimens (Table 1).

Further investigation of the disease excluded autoimmune causes (both Islet Cell Cytoplasmic Autoantibodies, ICCA, and Glutamic Acid Decarboxylase Autoantibodies, GADA, were negative) and revealed the following HLA haplotype: HLA-A*02, *24; HLA-B*07, * 08; HLA-C*04, * 07; DRB1*03, * 13; DQB1*02, * 06.

**Figure 1:**
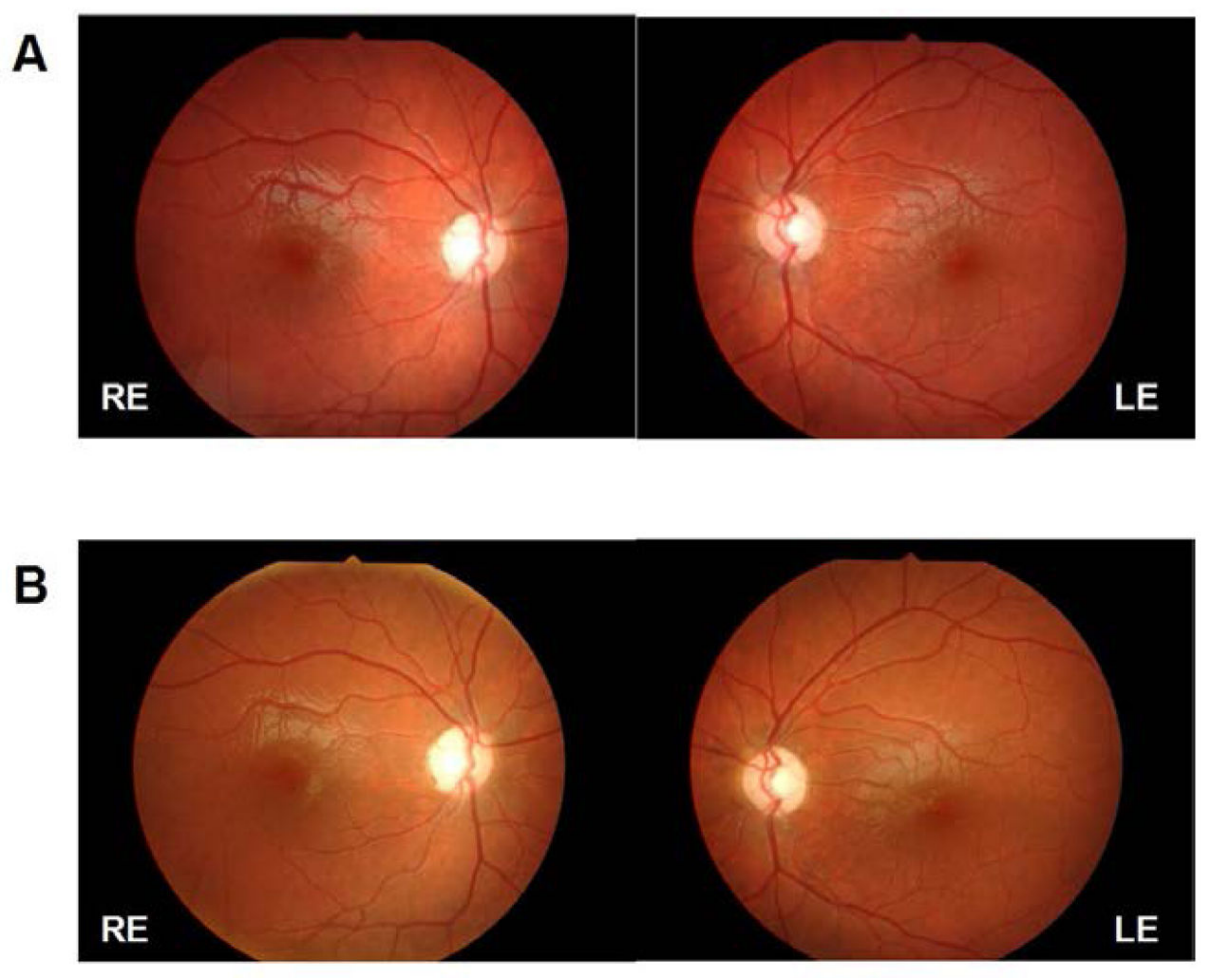
Retinographies at age 10 (A) and at age 16 (B), showing severe optic atrophy with no retinal changes suggestive of diabetic retinopathy. RE = right eye; LE: left eye.

His learning disabilities and general pediatric exam suggested a potential visual impairment, which prompted an evaluation by ophthalmology. At age 6, the patient presented with best-corrected visual acuity (BCVA) of 6/20 (3/10), bilateral optic nerve head pallor (Figure 1) and no other retinal abnormalities. The presence of bilateral optic atrophy associated with non-auto immune diabetes suggested a clinical diagnosis of Wolfram Syndrome (WS).

At age 8, nocturnal enuresis became frequent and ultrasonography suggested neurogenic bladder. A deteriorated performance at school was also noted due to problems in speaking (immature speech and difficulties in articulation and phonological processes), reading and interpreting, leading to his failing to pass to the next school year. His intelligence quotient (IQ) was evaluated with the Wechsler Intelligence Scale for Children (WISC-III) and determined to be 64 (an IQ between 50 and 69 is considered “borderline mental functioning” in this testing conditions).

At age 16, the patient had incomplete pubertal development with testicular atrophy associated with increased FSH levels, normal LH and normal total testosterone levels (Table 1). His height and weight were 1.65 m (5.41 ft; percentile P 10–25) and 64.5 kg (142.2 lbs; P 50–75), respectively. Regarding his metabolic status, abdominal lipodystrophy was evident and glycemic control remained highly inadequate (HbA1c 8.8 – 9%) under treatment with a 1.5 U/kg daily dose of insulin. His insulin sensitivity factor was 20 g/dl and his insulin/carbohydrates ratio was 5G. Blood pressure was 119/63 mmHg.

During the 10 years of follow-up, the patient underwent periodic multidisciplinary assessments; inadequate metabolic control was observed throughout these evaluations with HbA1c ranging from 8.6 to 9% despite multiple attempts with different therapeutic combinations and nutritional strategies.

Ophthalmological assessments revealed a progressive deterioration in BCVA [from 6/20 (3/10), at age 6, to 6/125 (1/20), at age 16] associated with continued atrophy of the optic disc (Figure 1) and significant functional impairment in visual field (Goldmann) and electrophysiological testing. Visual evoked potentials (VEP), an electrophysiological test that measures conductance of electrical impulse from the optic nerve to the brain, were significantly impaired showing increased latency and decreased amplitude of the P100 wave, especially in the left eye. However, no associated changes were noted in the full field ERG. These findings are suggestive of a significant and isolated defect at the level of the optic nerve.

The patient also underwent two audiometry exams at age 10 and 12 that were normal for all hearing frequencies. He did not develop any symptoms suggestive of diabetes insipidus or diabetic vascular complications, such as diabetic retinopathy, nephropathy or neuropathy. His thyroid function and cortisol values were within the normal range of values (Table 1).

Genetic analyses identified two sequence variants in homozygosity in the *WFS1* gene of our patient, who is the second child of a Self-reportedly non-consanguineous couple. The presence of non-consanguinity, however, could not be accurately determined because the father and the family members from the older generation refused to undergo genetic testing.

The sequence variants identified were the following:

1. A novel missense variant c1066T>C (pSer356Pro), in exon 8;
2. A previously described variant c482G>A (pArg161Gln), in exon 5, initially associated with low frequency sensorineural hearing loss (LFSNHL) [15] and later described in the 1000 Genomes Project [16] and interpreted as benign by Shearer et al. [17].

**Figure 2:**
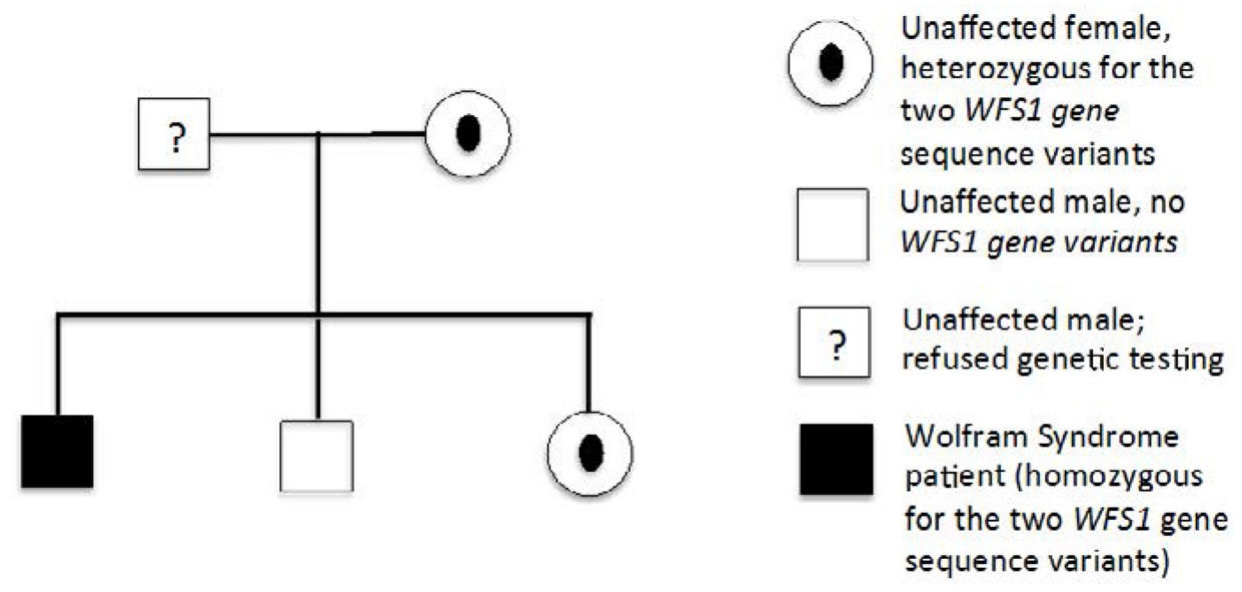
Segregation of the WFS1 gene variants (c1066T>C and c482G>A) in the nuclear family of the patient.

His mother and his 19-year-old sister were heterozygous for the same sequence variants in the *WFS1* gene, while the 11-year-old brother did not present any variations (Figure 2). These findings highly suggest that (1) both sequence variants must be located on the same chromosome (haplotype) and that (2) the presence of the two haplotypes in homozygosity is the cause for Wolfram syndrome in this family.

## Discussion

Wolfram syndrome, also known as DIADMOAD, is a rare autosomal recessive neurodegenerative disease that typically includes clinical features of insulin-dependent diabetes, diabetes insipidus, optic nerve atrophy and deafness that progress during the patient’s lifetime [2] (Table 2). The minimum diagnostic criteria are the presence of diabetes mellitus and optic nerve atrophy, which usually develop during the first decade [18].

The patient in this study came to our attention clinically due to a diagnosis of insulin dependent diabetes at the age of 6 which was later found to be non-autoimmune. This finding is quite unusual as 70–80 % of the type 1 Diabetes cases are initially positive for ICCA and GADA antibodies [19]. The diabetes in the patient was very challenging to manage and despite multiple therapeutic strategies, HbA1c levels were always above the desired values. He also presented prominent learning disabilities that were partly due to his visual impairment.

The presence of insulin dependent diabetes with peculiar features, such as non-autoimmune diabetes and difficult metabolic control, associated with visual impairment, or very significant learning disabilities, which may be masking a profound visual disorder, should prompt the clinician to consider Wolfram syndrome as a possible diagnosis. These patients need to be evaluated and managed by a multidisciplinary team to maximize their quality of life. Support and knowledge about the condition must be provided to their families, including information about prognosis; the mortality rate is very high with 60% of the patients dying by the age of 35 [20].

After establishing a clinical diagnosis of WS from the simultaneous presence of non-autoimmune diabetes mellitus and optic nerve atrophy, genetic testing was offered to our patient and his family. Two sequence variants were identified in homozygosity in the *WFS1* gene (exons 5 and 8):

The novel variation c1066T>C (pSer356Pro) is a missense variant predicted to be pathogenic (by MutationTaster and PolyPhen-2) and likely benign (by PROVEAN and SIFT). Mutations in the vicinity codons (350 and 361) have been described in association with Wolfram syndrome. Since this is a highly conserved residue, except in *Drosophila*, and family segregation is compatible, we interpret this variant as likely to be pathogenic.

The other sequence variant (c482G>A) has initially been reported to confer a 50% risk of developing autosomal dominant non-syndromic low-frequency hearing loss and has later been described in association with Wolfram syndrome [15,17]. Interestingly, our patient did not present any evidence of hearing impairment over the ten years of follow-up (Table 1).

To facilitate the diagnosis of WS within the type 1 diabetic population, Ehrlich and Fishman suggested in 1986 that certain HLA haplotypes could be of interest, (HLA DR2 had a higher prevalence in WS patients) [20]. Pinelli challenged this notion in 1987, claiming that the higher prevalence of specific HLA subtypes in the WS patient population was more likely a reflection of the genetic heterogeneity of the population from which those cases had arisen [21].

The work by Marshall et al. [2] showed that diabetes mellitus and optic nerve atrophy were the most common (94%) and earliest features to develop in young patients with WS [2]. Neurogenic bladder and dilations in the renal outflow tract are common in WS patients in their third decade of life. Our patient developed symptoms of enuresis due to a neurogenic bladder quite early, at the age of 8.

Marshall et al. reported that enuresis, nocturia and post-void residual bladder volume were present in 22%, 17% and 45%, respectively, of their young WS patient cohort [2].

Earlier studies have suggested that metabolic control is more easily attained in WS patients when compared to regular type 1 diabetic populations [22]. This was not observed in our patient where glycemic control proved to be incredibly challenging despite multiple attempts of therapeutic plan optimization and confirmed compliance with treatment.

A genetic variant in the *WFS1* human gene has been shown to determine impaired glucagon-like peptide-1-induced insulin secretion [23]; it is possible that the novel *WFS1* sequence variant identified in our patient plays a particularly disruptive role in beta cell functioning, contributing to profound impairment in insulin production, secretion or sensitivity.

Surprisingly, despite 10 years of highly inadequate metabolic control (HbA1c ranging from 8 to 9%) there was no evidence of diabetic retinopathy (DR) or other complications in our patient. Most clinical studies suggest that WS patients are relatively protected from developing diabetic microvascular complications when compared to regular type 1 diabetics, however the cause for this protection remains elusive [24,25]. Diabetes severely reduces metabolic supply to the retina, thereby generating a metabolic mismatch that drives development and progression of diabetic retinopathy (DR) [26]. It is possible that the protection against DR reported in WS patients is associated with premature retinal ganglion cell (RGC) death due to the optic atrophy that develops early on (RGC form the optic nerve) with consequent reduction in retinal metabolic demand. This potentially reduces the metabolic mismatch and leads to a better overall retinal energy status, thus eliminating the pathogenic stimulus that drives development and progression of DR.

Signs of hypogonadism are common in patients with WS and are usually attributed to hypothalamic or pituitary dysfunction [2]. The testicular atrophy and hormonal profile (high FSH with normal LH and testosterone) observed in our patient are consistent with a Sertoli-cell-only syndrome, also known as germinal cell aplasia [27]. These findings are consistent with animal studies reporting that *WFS1* deficient male mice show impaired fertility with significantly reduced number of spermatogonia and Sertoli cells [28]. As wolframin is involved in calcium homeostasis and in preventing ER stress, Haghighi et al. [29] suggested that mutations in the *WFS1* gene can disrupt ion homeostasis, affecting Sertoli cell development, sperm maturation and function, and ultimately reducing fertility [29].

## Conclusion

In the present study we report a 16 year-old patient with Wolfram syndrome with a therapeutically challenging form of non-autoimmune diabetes associated with a novel sequence variant in the *WFS1* gene.

Over the 10-year follow-up period by a multidisciplinary hospital team, our patient developed: (1) insulin-dependent diabetes that was difficult to control metabolically; (2) a profound visual deficit due to progressive optic nerve atrophy; (3) enuresis associated with neurogenic bladder; and (4) hypogonadism.

Interestingly, and in contrast to what would have been expected from his genotype, he did not develop hearing loss or diabetes insipidus. These findings reiterate the current notion that genotype-phenotype correlations are not clear in WS [6] and suggest that functional studies assessing interactions of different sequence variants and/or mutations in the *WFS1* gene may hold the key to a more precise understanding of the pathophysiology of this devastating syndrome.

## Figures and Tables

**Table 1:** Evolution of analytical parameters in our patient.

	Age 16	Age 14	Age 13	Age 6	Reference values
**Fasting blood glucose (mg/dl)**	183	272	198	352	70-105
**Urea (mg/dl)**	25	42	32	51	16.7-45.4
**Creatinine (mg/dl)**	0.8	0.9	0.7	0.5	0.7-1.3
**Uric acid (mg/dl)**	4.4	5.6	5.2	N/A	3.5-7.2
**Total cholesterol (mg/dl)**	130	142	133	N/A	< 200
**HDL (mg/dl)**	36	47	45	N/A	> 60
**LDL (mg/dl)**	85	82	72	N/A	< 100
**Triglycerides (mg/dl)**	44	63	79	N/A	< 150
**TSH (uUI/ml)**	2.54	2.33	2.09	4.52	0.35 -5.50
**Free T4 (ng/dl)**	1.12	1.24	1.07	1.36	0.89-1.76
**Cortisol (ug/dl)**	19.8	15.7	14.1	19.1	3.7 – 19.4
**Insulin (uU/ml)**	19.5	N/A	N/A	N/A	2.6-24.9
**C peptide (ng/ml)**	0.3	N/A	N/A	N/A	1.1 – 4.4
**LH (mUI/ml)**	6.20	7.92	3.88	N/A	1.14 – 8.75
**FSH (mUI/ml)**	22.24	31.95	16.09	N/A	1.37 – 8.75
**Estradiol (pg/ml)**	19	N/A	N/A	N/A	0
**Total testosterone (ng/dL)**	402	410	377	N/A	166-811
**Glucose (urine) mg/dl**	1000	N/A	N/A	N/A	0
**Glomerular filtration rate (MDRD-4) ml/min**	137.06	N/A	N/A	N/A	> 60

**Table 2:** Clinical features present in our patient in comparison to those commonly reported.

Clinical features	Typical WFS1 cases	WFS1 our case
**Diabetes mellitus**	X	X
**Optic atrophy**	X	X
**Sensorineural Hearing Loss**	x	
**Diabetes insipidus**	X	
**Neurological disorders**	X	x
**Genito-urinary tract problems**	X	
**Hypogonadism**	X	x
